# Xpert MTB/RIF assay for diagnosis of extrapulmonary tuberculosis in children: a systematic review and meta-analysis

**DOI:** 10.1186/s12879-019-4745-1

**Published:** 2020-01-06

**Authors:** Young Seok Seo, Ji-Man Kang, Dong Soo Kim, Jong Gyun Ahn

**Affiliations:** Department of Pediatrics, Severance Children’s Hospital, Yonsei University College of Medicine, 50-1 Yonsei-ro, Seodaemun-gu, Seoul, 03722 South Korea

**Keywords:** Extrapulmonary tuberculosis, Xpert MTB/RIF, Child, Meta-analysis

## Abstract

**Background:**

The Xpert® MTB/RIF assay (Xpert; Cepheid, Sunnyvale, CA, USA) is a cartridge-based nucleic acid amplification assay for rapidly diagnosing tuberculosis and assessing antibiotic sensitivity. Although previous evidence supports the use of Xpert for diagnosing extrapulmonary tuberculosis (EPTB) in adults, information regarding the accuracy of Xpert for EPTB only in children is lacking. This meta-analysis was performed to assess the accuracy of Xpert for detecting EPTB in children.

**Methods:**

We searched the MEDLINE, EMBASE, and Cochrane Infectious Diseases Group Specialized Register from January 1, 2010 to July 16, 2019 for studies of the diagnostic performance wherein Xpert was analyzed against cultures or composite reference standards for < 18-year-old children with EPTB.

**Results:**

In only pediatric studies, 8 studies including 652 samples were selected. The pooled sensitivity and specificity of Xpert for all samples were 71% (95% CI 0.63–0.79) and 97% (95% CI 0.95–0.99), respectively. The area under the summary receiver operating characteristic (sROC) curve was 0.89. For lymph node tissues or aspirates, the pooled sensitivity and specificity of Xpert were 80% (95% CI 0.70–0.88) and 94% (95% CI 0.89–0.97), respectively; for cerebrospinal fluid (CSF), these values were 42% (95% CI 0.22–0.63) and 99% (95% CI 0.95–1.00), respectively.

**Conclusion:**

Overall, Xpert displayed high specificity but modest sensitivity across various samples for diagnosing pediatric EPTB compared to the composite reference standard. Xpert sensitivity varied with the sampling site and was especially lower in CSF samples. Positive Xpert results may be considered to indicate a presumptive case of pediatric EPTB, whereas negative test results indicate that the possibility of pediatric EPTB should not be excluded.

## Background

Tuberculosis (TB) is a serious threat to global public health and the leading cause of death from a single infectious disease worldwide, surpassing the rates of malaria and HIV/AIDS. Globally, there were an estimated 10.0 million incident cases of TB in 2018, with approximately 1 million (11%) among children < 15 years. However, the mortality rate was higher in children aged < 15 years, accounting for 14% of total deaths, which is greater than that in incident cases, suggesting poorer access to diagnosis and treatment [1].

Extrapulmonary tuberculosis (EPTB) refers to TB occurring within a location in the body other than the lungs (e.g., meninges, lymph nodes, pleura, abdomen, genitourinary tract, skin, joints, and bones) [2]. EPTB is estimated to account for 8–24% (15% of the 7.0 million incident cases on average) of all TB infections worldwide [1]. These numbers vary in accordance with specific risk factors in certain regions, such as age, sex, concurrent HIV infection, and underlying comorbidities [1, 3]. EPTB commonly occurs in children and HIV-infected individuals [4].

To diagnose EPTB, samples should be obtained from sites of suspected infection and cultured. The diagnosis of pediatric EPTB remains challenging because clinical specimens are potentially inaccessible for appropriate sampling and require invasive diagnostic procedures [5]. Furthermore, 8–12 weeks are required to obtain the results through culturing, thus delaying treatment [6]. Because of these diagnostic challenges among children, the incidence of pediatric EPTB is likely underestimated [7].

The Xpert® MTB/RIF assay (Xpert; Cepheid, Sunnyvale, CA, USA) is a cartridge-based nucleic acid amplification assay for rapid TB diagnosis and rapid antibiotic sensitivity analysis. Currently, Xpert is used as a rapid assay for TB diagnosis as recommended by the World Health Organization (WHO) [1]. Since 2013, Xpert has also been recommended for diagnosing TB meningitis and TB lymphadenitis in children [8]. Several systematic reviews have been conducted to determine the diagnostic accuracy of Xpert for EPTB in both pediatric and adult populations; however, no studies have specifically evaluated for children [5, 9–16]. Therefore, data regarding the accuracy of Xpert exclusively among children are unavailable. We conducted a systematic review and meta-analysis to assess the diagnostic accuracy of Xpert for detecting EPTB among children.

## Methods

### Data sources and search strategies

We searched the MEDLINE, EMBASE, and Cochrane Infectious Diseases Group Specialized Register. Our last search was carried out on July 16, 2019. Furthermore, we manually reviewed the bibliographies of the included articles. The primary search terms were “Xpert,” “GeneXpert,” “Cepheid,” “MTB/RIF,” and “Tuberculosis.” The search methodology applied for each database is shown in Additional file 1. The bibliography was screened for full-length research articles in all languages. Moreover, we reviewed the full-text to select articles describing the exclusive analysis of pediatric EPTB.

### Eligibility criteria

The following inclusion criteria were used: (1) studies using Xpert as a diagnostic tool for detecting EPTB compared to a reference standard in each study, with all non-respiratory samples (i.e., lymph node aspirate or tissue, CSF, pleural fluid, etc.); (2) studies evaluating the diagnostic performance of Xpert; and (3) studies providing pediatric (0–18 years) data. Studies were included regardless of HIV infection status.

We excluded reviews, letters, editorials, expert opinions, animal experiments, and studies that only presented an abstract. Studies that did not include separate pediatric data were also excluded. We attempted to include all types of EPTB samples; however, studies reporting the use of gastric lavage samples were excluded because they were intended to diagnose pulmonary TB. Studies including samples from fewer than five patients and studies with no or insufficient data to construct a 2 × 2 contingency table to determine sensitivity and specificity were also excluded. If data were obtained in more than one article from the same author, the article with the most data was selected.

### Study selection

Two review authors (YS Seo and JK Ahn) independently assessed the titles and abstracts in accordance with the inclusion and exclusion criteria, followed by a full-text review of the selected studies. Discrepancies regarding the inclusion of articles between the two authors were resolved by the third author (DS Kim).

### Composite reference standard (CRS)

To compare the accuracy of Xpert, mycobacterial culturing or a CRS was used as a reference standard. The CRS was defined by the authors of each study. Because of the paucibacillary characteristics of extrapulmonary TB, the clinical diagnosis of TB was also included. The CRS included histopathological, smearing, and clinical response analysis to treatment with anti-TB therapy along with culturing.

### Quality assessment

Qualitative assessment was performed using the Quality Assessment of Diagnostic Accuracy Studies-2 (QUADAS-2) tool [17]. All eligible studies were evaluated based on four domains: patient selection, index test, reference standard, and flow and timing. Each domain was assessed in terms of the risk of bias, and the first three domains were assessed in terms of concerns regarding applicability.

### Statistical analysis

We determined the sensitivity and specificity of Xpert with 95% confidence intervals compared to culturing or the CRS. To assess the heterogeneity among studies, the chi-square test was performed. Heterogeneity was defined as a *p*-value of < 0.10. In case of heterogeneity, different thresholds were considered to influence sensitivity and specificity. To assess the presence of a threshold effect, Spearman’s ρ correlation analysis was performed with ρ > 0.6 indicating a threshold effect. The sensitivities and specificities of Xpert in each study were determined and subjected to meta-analysis with a bivariate random-effects model. We plotted the summary receiver operating characteristic (sROC) curve with this model. R-package mada (version 3.5.1.) was used to generate forest plots and an ROC curve.

## Results

### Identified studies

**Fig. 1:**
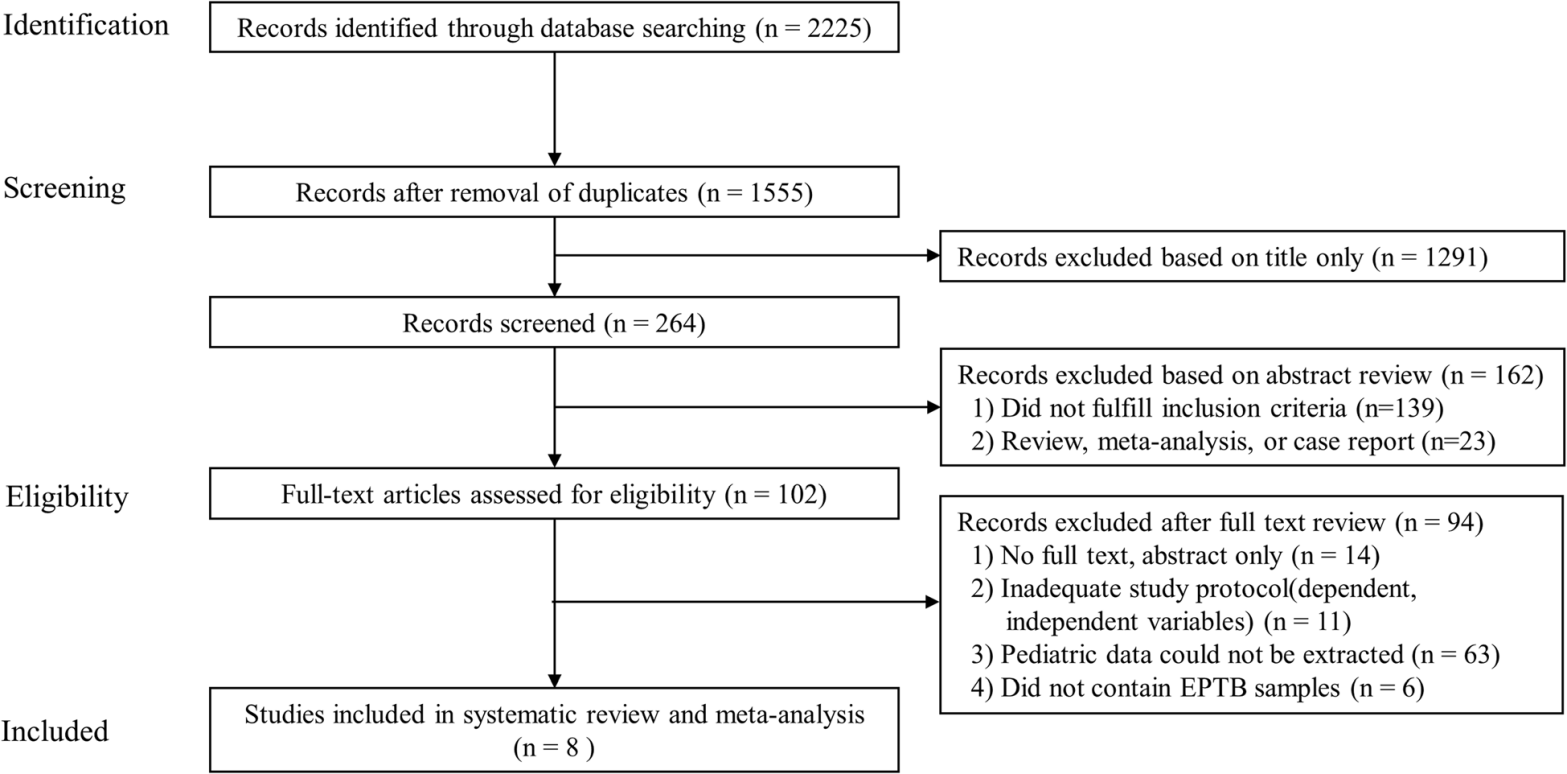
Flow diagram showing the protocol for study selection. EPTB, extrapulmonary tuberculosis

Figure 1 shows the protocol for the screening of articles. Of the 2225 articles obtained from MEDLINE (*n* = 844), EMBASE (*n* = 1180), and Cochrane (*n* = 201), 670 duplicates were excluded. After screening the titles, 1291 studies were excluded. After screening the abstracts, 162 studies were excluded for the following reasons: 139 studies did not fulfill the inclusion criteria and 23 studies were excluded because they were review studies, meta-analysis studies, or case reports. After reviewing the full-text of the remaining 102 studies, 94 studies were excluded for the following reasons: 14 studies included only abstracts, 6 studies did not contain EPTB samples, 11 studies had inadequate study protocols, and 63 studies did not separate pediatric data. Finally, we identified 8 studies that included 652 samples.

### Study characteristics

Table 1 summarizes the characteristics of the included studies. Five studies were conducted in Africa [18–22], 1 study in India [23], 1 study in South Korea [24], and 1 in Italy [25]. Two studies were retrospective [24, 25] and the remaining 6 were prospective [18–23]. All articles were written in English. Sample numbers varied in accordance with the studies. In total, 277 lymph nodes, 218 CSF samples, 20 pleural fluid samples, and 137 musculoskeletal samples were reviewed.

**Table 1:** Characteristics of the included studies

Study	First author	Year	Country	Setting	Age	Study design	HIV%	Culture reference standard	Composite reference standard (CRS)	Total samples (n)	Specimen type
1	Bholla, M.	2016	Tanzania	Primary care Center	8 week – 16 years	Prospective	20	MGIT	Cytology and/or Culture	75	Lymph node
2	Coetzee, L.	2014	South Africa	Tertiary care center	< 13 years	Prospective	8.3	MGIT	Cytology and/or Culture	72	Lymph node
3	Das, A.	2019	India	Tertiary care center	4 month – 14 years	Prospective	1.75	MGIT/LJ	Culture	57	Lymph node (*n* = 6), CSF (*n* = 51),
4	Held, M.	2016	South Africa	Tertiary care center	< 13 years	Prospective	10	MGIT	Culture or histology	109	Bone and joint tissue
5	Kim, YW.	2015	South Korea	Tertiary care center	0–18 years	Retrospective	0.8	MGIT	Culture	92	Lymph node (*n* = 30) CSF (*n* = 14) Joint fluid (*n* = 28) Pleural fluid (*n* = 20)
6	Solomons, R.S.	2015	South Africa	Tertiary care center	3 month – 13 years	Prospective	11	MGIT	Clinical TBM reference standard	101	CSF
7	Tortoli, E.	2012	Italia	Tertiary care center	0–18 years	Retrospective	10	MGIT/LJ	Histopathology /Improvement on ATT	132	Lymph node (*n* = 89), CSF (*n* = 43),
8	Vadwai	2011	South Africa	Tertiary care center	< 13 years	Prospective	3	MGIT	Histopathology /Improvement on ATT	14	Lymph node (n = 5), CSF(*n* = 9)

*ATT* anti-tubercular treatment; *CSF* cerebrospinal fluid; *LJ* Löwenstein-Jensen culture; *MGIT* mycobacterial growth indicator tube; *TBM* tuberculous meningitis

### Quality assessment

**Fig. 2:**
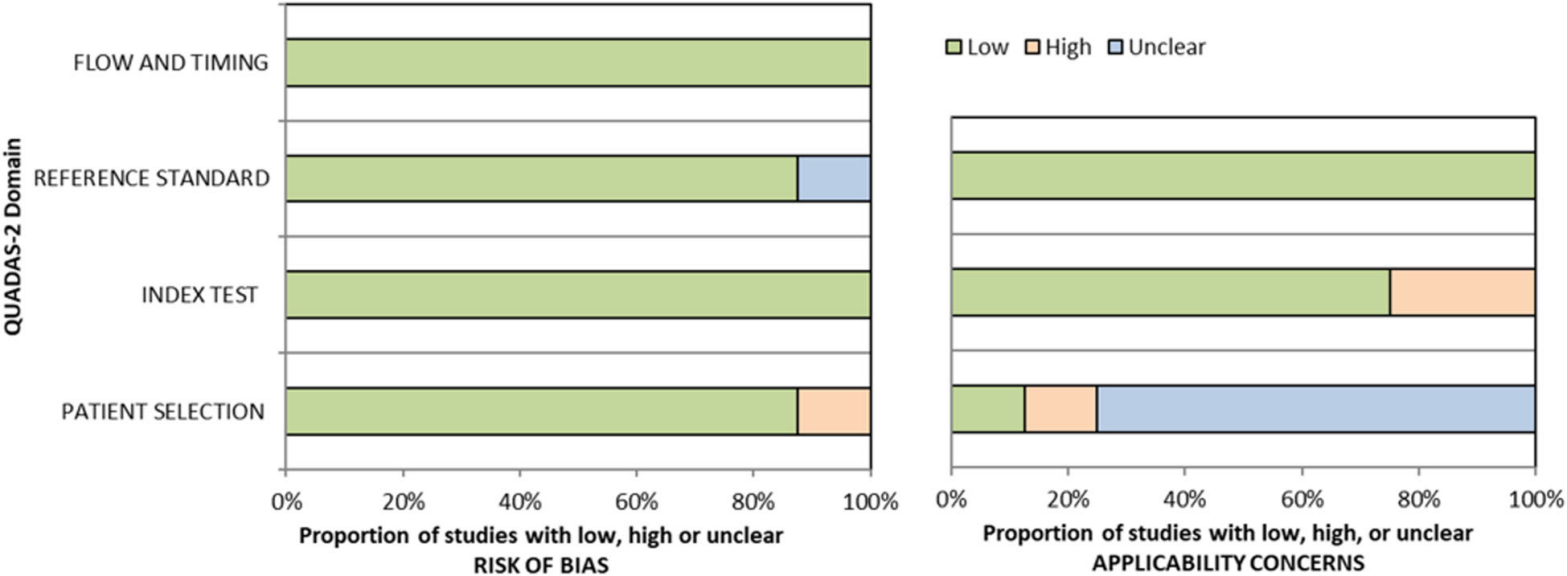
Quality assessment based on Quality Assessment of the Diagnostic Accuracy Studies-2 (QUADAS-2) guidelines. Graphical representation of the risk of bias and applicability concerns

Quality assessment was performed using QUADAS-2, as summarized in Fig. 2. In the patient selection domain, one study reported a high risk of bias, wherein patients were selected through convenience [18]. Other studies reported a low risk of bias. Regarding applicability, one study [18] had low concern because patients were assessed in a local hospital; another study [20] revealed high concern because only inpatients were evaluated at a tertiary-care center [18, 20]. Other studies reported unclear concern because of a lack of enough information regarding the clinical setting [19, 21–25]. For the index test and reference standard, the included studies generally had a low risk of bias and low applicability concerns.

### Meta-analysis for diagnostic accuracy of Xpert

**Fig. 3:**
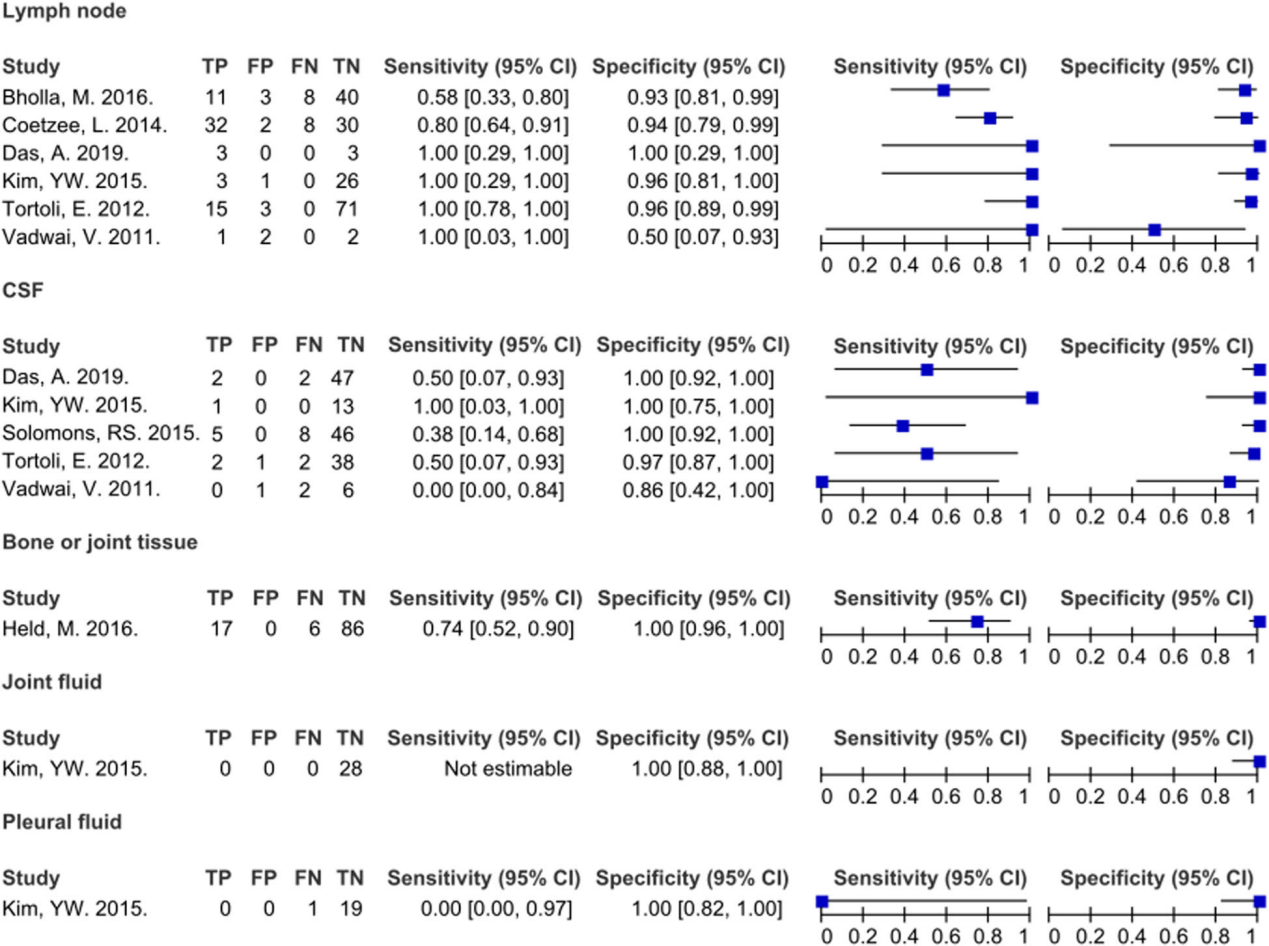
Forest plot of the sensitivity and specificity of Xpert in diagnosing extrapulmonary tuberculosis in comparison with a composite reference standard in accordance with the study and specimen type. TP, true-positive; FP, false-positive; FN, false-negative; TN, true-negative

**Fig. 4:**
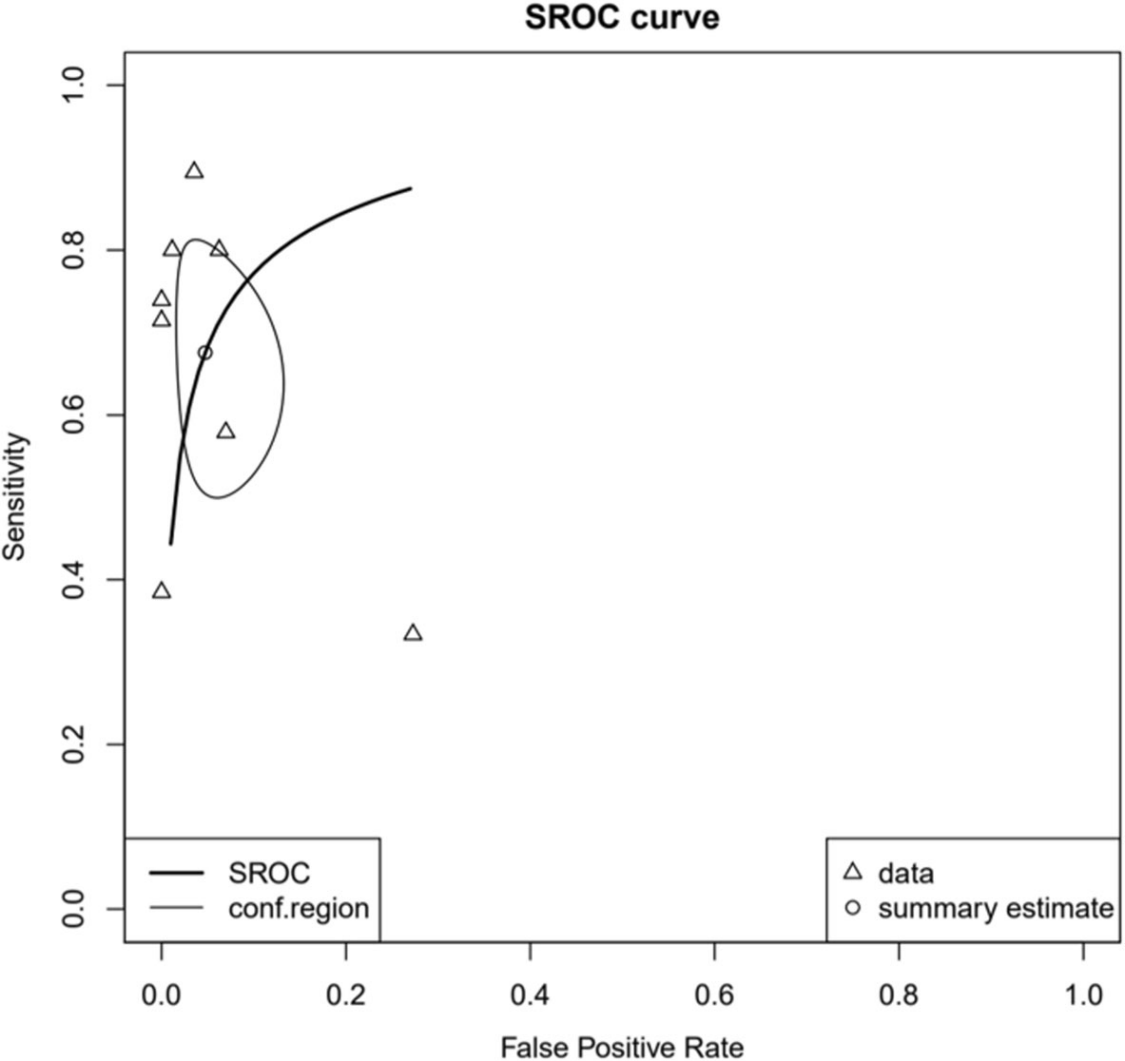
Hierarchical summary receiver operating characteristic (HSROC) curve of the diagnostic accuracy of Xpert® MTB/RIF assay (Xpert) for extrapulmonary tuberculosis (EPTB). Summary points of the sensitivity and specificity, HSROC curve, and 95% confidence intervals. The area under the curve of the HSROC for Xpert was 0.89

The 8 studies were evaluated as described above (Fig. 3). Regardless of the sample type, the pooled sensitivity and specificity of all samples were 71% (95% CI 0.63–0.79) and 97% (95% CI 0.95–0.99), respectively. The area under the ROC curve was 0.89 (Fig. 4). High heterogeneity was confirmed through chi-square analysis for both sensitivity and specificity. However, it was difficult to assign statistical significance to the data because of the heterogeneity among sample types. Therefore, each sample was divided into subgroups.

### Detection of lymph node TB

Six studies used Xpert to analyze lymph node samples obtained by fine-needle aspiration biopsy (FNAB) or lymph node biopsy rather than a CRS [18, 19, 22–25]. The pooled sensitivity and specificity of the lymph node samples were 80% (95% CI 0.70–0.88) and 94% (95% CI 0.89–0.97), respectively. The area under the ROC curve was 0.92. High heterogeneity was confirmed using the chi-square test for specificity.

### Detection of TB meningitis

Five studies used Xpert to analyze CSF samples rather than a CRS [21–25]. The pooled sensitivity and specificity of the CSF samples were 42% (95% CI 0.22–0.63) and 99% (95% CI 0.95–1.00), respectively. The area under the ROC curve was 0.57. High heterogeneity was confirmed through chi-square analysis for specificity.

### Comparison with other published meta-analyses

Nine meta-analyses have been performed to assess the accuracy of Xpert for detecting EPTB (Table 2) [5, 9–16]. These reviews evaluated data from both children and adult populations; however, no studies analyzed children alone. In these analyses, the diagnostic accuracy of Xpert in lymph node samples showed sensitivities ranging from 83 to 96% and specificities ranging from 86 to 94% (80.2 and 94.0% in this study) [5, 10, 12, 13, 16]. In CSF samples, the sensitivities ranged from 69 to 85% and specificities ranged from 97 to 100% (41.7 and 98.7% in this study) [5, 10, 12, 13]. In the pleural fluid samples, the sensitivities ranged from 34 to 51.4% and specificities ranged from 98 to 99% [5, 10, 12–14]. In bone or joint tissue specimens, sensitivities and specificities ranged from 84 to 91.8% and 82 to 98%, respectively [10, 15]. In bone or joint pus specimens, the sensitivity was 82% and specificity was 99% [15]. In the joint fluid specimens, sensitivity and specificity were 97.2% and from 90.2%, respectively [10]. However, in this study, meta-analysis of pleural TB and bone or joint TB could not be performed because of the small number of studies.

**Table 2:** Comparison of published meta-analyses of Xpert® MTB/RIF for diagnosis of extrapulmonary tuberculosis

Author/Year	Search period	Groups of children included	Number of studies included in the meta-analysis	Number of specimens included	Specimen types	Accuracy of Xpert				
						All samples	Lymph node TB	TB meningitis	Pleural TB	Bone or joint TB
Chang, 2012	Up to Oct 1, 2011	No classification by age	7	1058	Multiple forms combined	Sensitivity 80.4%; Specificity 86.1%	Not reported	Not reported	Not reported	Not reported
Denkinger, 2014	Up to Oct 15, 2013	10 studies included children	18	4461	Lymph node, CSF, Pleural fluid	Sensitivity 97.4%; Specificity not reported	Sensitivity 83%; Specificity 94%	Sensitivity 81%; Specificity 98%	Sensitivity 46%; Specificity 99%	Not reported
Maynard-Smith, 2014	Up to Nov 6, 2013	3 studies enrolled only children, 3 included all age-groups	27	6026	Lymph node, CSF, Other forms	Median sensitivity 83% (IQR, 68–94%; median specificity 98% (IQR, 89–94%)	Sensitivity 96%; Specificity 93%	Median sensitivity 85% (IQR, 75–100%); Median specificity 100% (IQR, 98–100%)	Sensitivity 34%; Specificity 98%	Not reported
Penz, 2015	Up to Aug 15, 2014	No classification by age	36	9523	Lymph node, CSF, Other forms	Sensitivity 77%; Specificity 97%	Sensitivity 87%; Specificity 92%	Sensitivity 69%; Specificity 97%	Sensitivity 37%; Specificity 98%	Not reported
Sehgal, 2016	Up to Aug 31, 2015	No classification by age	24	2486	Pleural fluid	Pleural fluid samples only	Not reported	Not reported	Sensitivity 51.4%; Specificity 98.6%	Not reported
Li S, 2017	Up to June 20, 2015	No classification by age	26	Not reported	Multiple forms combined	Sensitivity 80%; Specificity 97%	Not reported	Not reported	Not reported	Not reported
Kohli, 2018	Up to Aug 7, 2017	30 studies included children	66	16213	Lymph node, CSF, Other forms	Varied across different types of specimens	Sensitivity 87.6%; Specificity 86.0%	Sensitivity 71.1%; Specificity 98.0%	Sensitivity 50.9%; Specificity 99.2%	Sensitivity 97.2% (fluid), 91.8%(tissue); Specificity 90.2% (fluid), 82.0% (tissue)
Yu G, 2019	Up to Jul 6, 2018	No classification by age	21	1629	Lymph node	Lymph node samples only	Sensitivity 84%; Specificity 91%	Not reported	Not reported	Not reported
Shen Y, 2019	Up to May 7, 2019	No classification by age	14	1884	Bone or joint samples	Bone and joint samples only	Not reported	Not reported	Not reported	Sensitivity 82% (pus), 84% (tissue); Specificity 99% (pus), 98% (tissue)
Current study	Up to Jul 16, 2019	Only samples taken from children were included	8	597	Lymph node, CSF, Other forms	Sensitivity 71.3%; Specificity 97.2%	Sensitivity 80.2%; Specificity 94.0%	Sensitivity 41.7%; Specificity 98.7%	Meta-analysis could not be performed	Meta-analysis could not be performed

*EPTB* extrapulmonary tuberculosis, *TB* tuberculosis, *IQR* interquartile range, *CSF* cerebrospinal fluid

## Discussion

The present study summarizes the overall performance of Xpert for diagnosing pediatric EPTB based on the currently available literature. Although previous systematic reviews analyzed data from both children and adult populations, no studies have reported distinct data for children [5, 9–16]. This study shows that Xpert has high specificity in pediatric EPTB, although its sensitivity is relatively lower and highly variable among specimen types.

In a recent meta-analysis reporting data primarily about adults, the pooled sensitivity varied among different types of specimens (83.1% in lymph node aspirates, 71.1% in CSF, and 94.6% in bone or joint tissue). However, the pooled specificity was relatively high between sample types (86% in lymph node aspirates, 98% in CSF, and 85.3% in bone or joint tissue) [10]. These data agree with the present results in that Xpert showed high specificity among various specimens in our study. Furthermore, the pooled sensitivity varied among the different types of specimens in this study. Overall, however, the sensitivity was lower among children than among adults, particularly in CSF samples (42% vs. 71%) [10]. In the case of musculoskeletal TB and pleural TB, the data were insufficient to carry out meta-analysis to determine the diagnostic accuracy of Xpert in pediatric populations, as two studies of musculoskeletal TB used different sample types (tissue vs. fluid), and only one study used pleural TB samples [20, 24]. However, these studies also displayed lower sensitivity in children than in adults, likely because the sample volume that can be collected from children is relatively lower than that from adults and because of the paucibacillary nature of EPTB in the former [21, 23, 26]. For CSF samples, a high sample volume was shown to increase the sensitivity of Xpert [27]. The total number of TB bacilli in the test sample plays an important role in the sensitivity of Xpert [27, 28], indicating that the sensitivity of Xpert in liquid samples may be lower than expected.

Since 2013, the WHO has recommended Xpert rather than conventional tests for the diagnosis of TB meningitis and TB lymphadenitis in children. As per the 2013 WHO data, the pooled sensitivity and specificity of lymph node TB in children were 86% (95% CI 0.65–0.96) and 81% (95% CI 0.54––0.93), respectively. In the case of TB meningitis, the pooled specificity was 95% (95% CI 0.81–0.99) and sensitivity could not be determined because of insufficient data [8]. To our knowledge, this is the first meta-analysis to evaluate the sensitivity of Xpert for TB meningitis only in pediatric populations. Our results suggest that negative Xpert results in children should be interpreted with caution with respect to ruling out pediatric TB meningitis. However, because TB meningitis is potentially lethal in children, the rapidity of Xpert explains why it should be used as an initial diagnostic test for TB meningitis despite its low sensitivity.

Recently, the next-generation Xpert MTB/RIF assay, Xpert MTB/RIF Ultra assay (Cepheid) (ULTRA), was developed; its limit of detection was enhanced by ~ 8-fold compared to the previous Xpert MTB/RIF assay and it includes a larger chamber and additional molecular targets [29]. In 2017, the WHO recommended the use of ULTRA as a replacement for Xpert MTB/RIF in all settings [30]. A single study of TB meningitis conducted in Uganda assessed the diagnostic accuracy of Xpert and ULTRA compared to CRS based on a positive CSF culture, Xpert, or ULTRA results. The sensitivity of detection of MTB for CSF was 95% for ULTRA (21 of 22) relative to 45% for Xpert MTB/RIF (10 of 22) [28]. ULTRA is expected to have higher sensitivity for EPTB. However, as discussed herein, pediatric EPTB samples showed lower sensitivity than adult samples. ULTRA would be helpful for the paucibacillary population, particularly in terms of diagnostic sensitivity. However, considering that the sensitivity of the previous version of Xpert is low in children and yielded different values among samples, further studies are required to determine the reliability of the negative results.

EPTB is common in children but difficult to diagnose because the sampling methods are invasive. A point-of-care ultrasound (POCUS) protocol (focused assessment with sonography for HIV-associated TB, FASH) is a noninvasive diagnostic tool developed to improve the diagnosis of EPTB in HIV-infected adults [31]. Although few studies have been conducted in children, POCUS has been evaluated for the diagnosis of pediatric EPTB, with some studies showing meaningful results in children [32]. A combination of various diagnostic methods such as Xpert and POCUS may improve the accuracy of diagnosing pediatric EPTB.

The present meta-analysis revealed high heterogeneity in patients with TB lymphadenitis and meningitis. Although the population was limited to those of the pediatric age and the samples were divided into subgroups, differences in the processing methods of samples and the small sample size may have resulted in high heterogeneity.

## Conclusion

In diagnosing pediatric EPTB, Xpert displayed high specificity regardless of the specimen type, but exhibited modest sensitivity, which varied among specimen types. Particularly, in CSF samples, Xpert displayed the lowest sensitivity compared to the CRS. Although positive Xpert results can be considered to indicate presumptive EPTB in children, EPTB cannot be ruled out based on negative test results. Future clinical trials are required to expand the evidence for using Xpert to diagnose pediatric EPTB with different forms of extrapulmonary specimens in various clinical settings.

## Supplementary information

**Additional file 1.** Study search strategy

## Data Availability

The data used in the present study are appropriately cited.

## Abbreviations

CI: Confidence interval
CRS: Composite reference standard
CSF: Cerebrospinal fluid
EPTB: Extrapulmonary tuberculosis
FNAB: Fine needle aspirationbiopsy
LN: Lymph node
ROC: Receiver operating characteristic
TB: Tuberculosis
ULTRA: Xpert MTB/RIF Ultra assay
